# PHYSICAL VERSUS COGNITIVE ACTIVITY FOR SUCCESSFUL AGING: BRAIN MAINTENANCE OR COGNITIVE RESERVE?

**DOI:** 10.1093/geroni/igz038.2307

**Published:** 2019-11-08

**Authors:** Kaitlin Casaletto, Kaitlin B Casaletto, Judy Pa, Sarah Tom, Miguel Arce-Renteria, Amal Harrati, Nicole Armstrong, Laura Zahodne

**Affiliations:** 1 University of California, San Francisco, San Francisco, California, United States; 2 UCSF, San Francisco, California, United States; 3 University of Southern California, Los Angeles, California, United States; 4 Columbia University, New York, New York, United States; 5 Stanford University, Stanford, California, United States; 6 NIH-NIA, Bethesda, Maryland, United States; 7 University of Michigan, Ann Arbor, Michigan, United States

## Abstract

Mechanisms by which physical (PA) and cognitive (CA) activities promote healthy cognitive aging are unknown. We examined independent contributions of PA and CA to “brain maintenance” (MRI markers of brain integrity) versus “cognitive reserve” (better cognition than predicted by brain integrity) in two independent samples of non-demented older adults (UCSF n=344; UCD n=482). In UCSF, only PA was positively associated with white matter (WM) integrity, while CA attenuated the relationship between WM and cognition. This pattern suggests PA supports brain maintenance, while CA contributes to cognitive reserve. In UCD, CA was positively associated with total gray matter volume; PA was positively associated with age-related WM integrity, and attenuated the association between WM and cognition. This indicates that both PA and CA support brain maintenance, with PA more strongly related to cognitive reserve. There may be preferential, but overlapping pathways by which PA and CA maintain age-related brain and cognitive health.

